# A Rare Case of Delayed Hypersensitivity Following COVID-19 Booster Necessitating Treatment With Dupilumab

**DOI:** 10.7759/cureus.37544

**Published:** 2023-04-13

**Authors:** Maryam Hanoodi, Kristin Logee DO

**Affiliations:** 1 Internal Medicine, Vassar Brothers Medical Center, Poughkeepsie, USA; 2 Rheumatolgy, Nuvance Health, Poughkeepsie, USA

**Keywords:** covid-19, delayed hypersensitivity reaction, dupilumab, covid-19 vaccine, atopic eczema

## Abstract

Following vaccination, patients can develop symptoms of eczema flare, which could range from mild skin irritation and urticaria to diffuse skin involvement. Delayed immunologic reactions have been described in association with the novel mRNA COVID‐19 vaccines and boosters. We report the case of an 83‐year‐old female who presented with widespread pruritic urticarial indurated papules on the arms, legs, and palms, sparing the face six months following the booster vaccine. She denied constitutional symptoms, new medications, recent illnesses, or new personal care products. Punch biopsy demonstrated acanthosis, spongiosis, and superficial and mild dermal perivascular lymphocytic infiltration with occasional eosinophils compatible with a dermal hypersensitivity reaction. The patient was admitted to the hospital due to the need for systemic steroids as well as IV antibiotics secondary to a superimposed bacterial skin infection in the setting of severe itching and skin injury; she was discharged on oral steroids with follow-up to dermatology and rheumatology. Delayed hypersensitivity reactions typically peak within four days following vaccination and may be observed with COVID-19 vaccines or boosters. However, reports remain limited, and people’s history of eczema should not preclude them from receiving a COVID-19 vaccine that is both safe and effective.

## Introduction

Eczema is a chronic inflammatory skin disease. It is caused by loss of function mutation of the filaggrin (FLG) gene. Eczema causes dry skin, severe pruritis commonly on the face, neck, and extensor surfaces in infants and children, and flexural surface in the adult population, sparing the axillary and groin region. According to the national eczema association, more than 31 million people in the United States have been affected [1]. Eczema can flare up as a local or disseminated vaccinal reaction that can develop in patients who recently received a live vaccine, like smallpox, causing what is called eczema vaccinatum (Kaposi varicelliform eruption) [2]. It can also be manifested as an extensive vesiculopustular rash with systemic illness [3]. In light of the above, a history of eczema has been considered one of the contraindications for vaccination in a non-smallpox emergency.

## Case presentation

**Figure 1:**
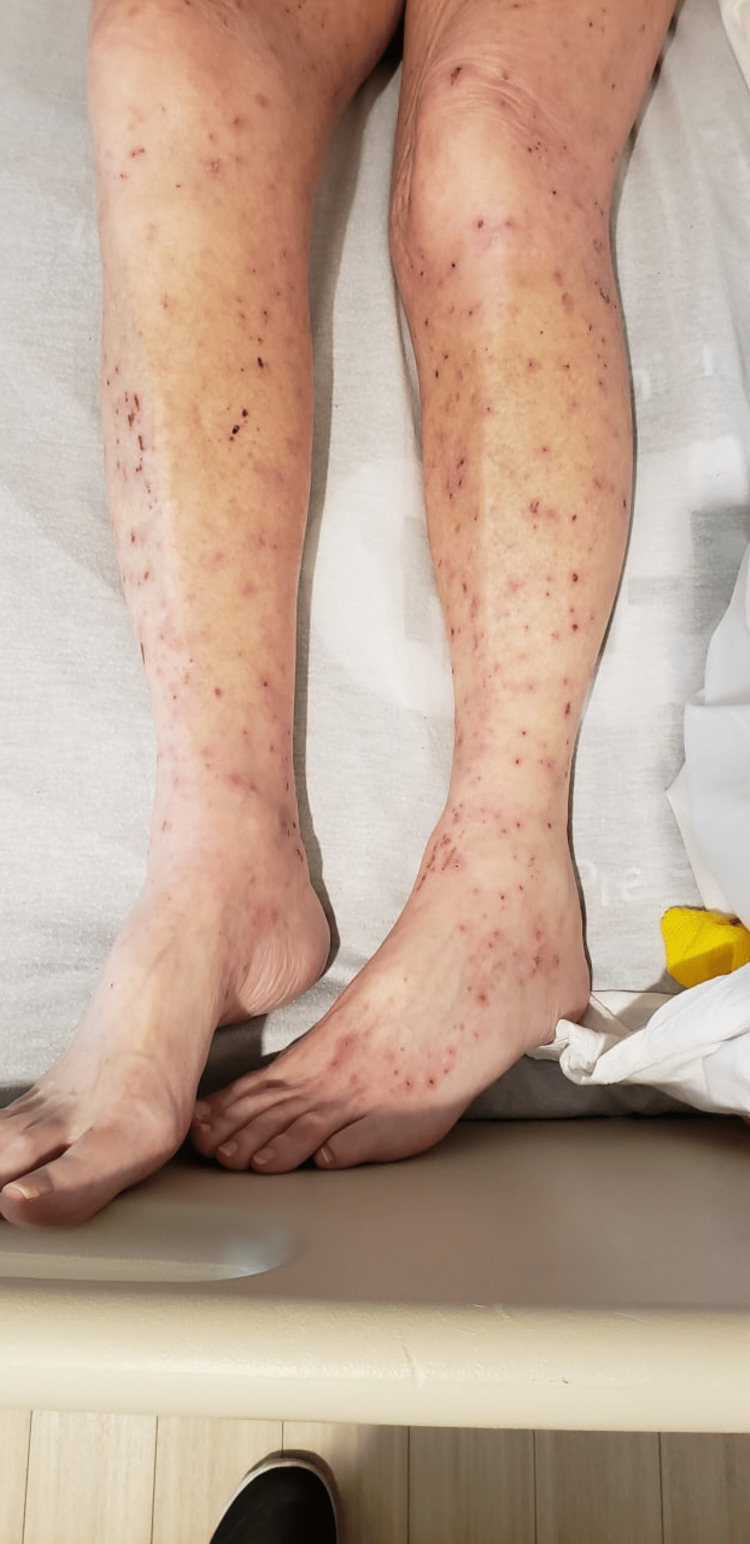
Urticarial papules on the lower extremities

**Figure 2:**
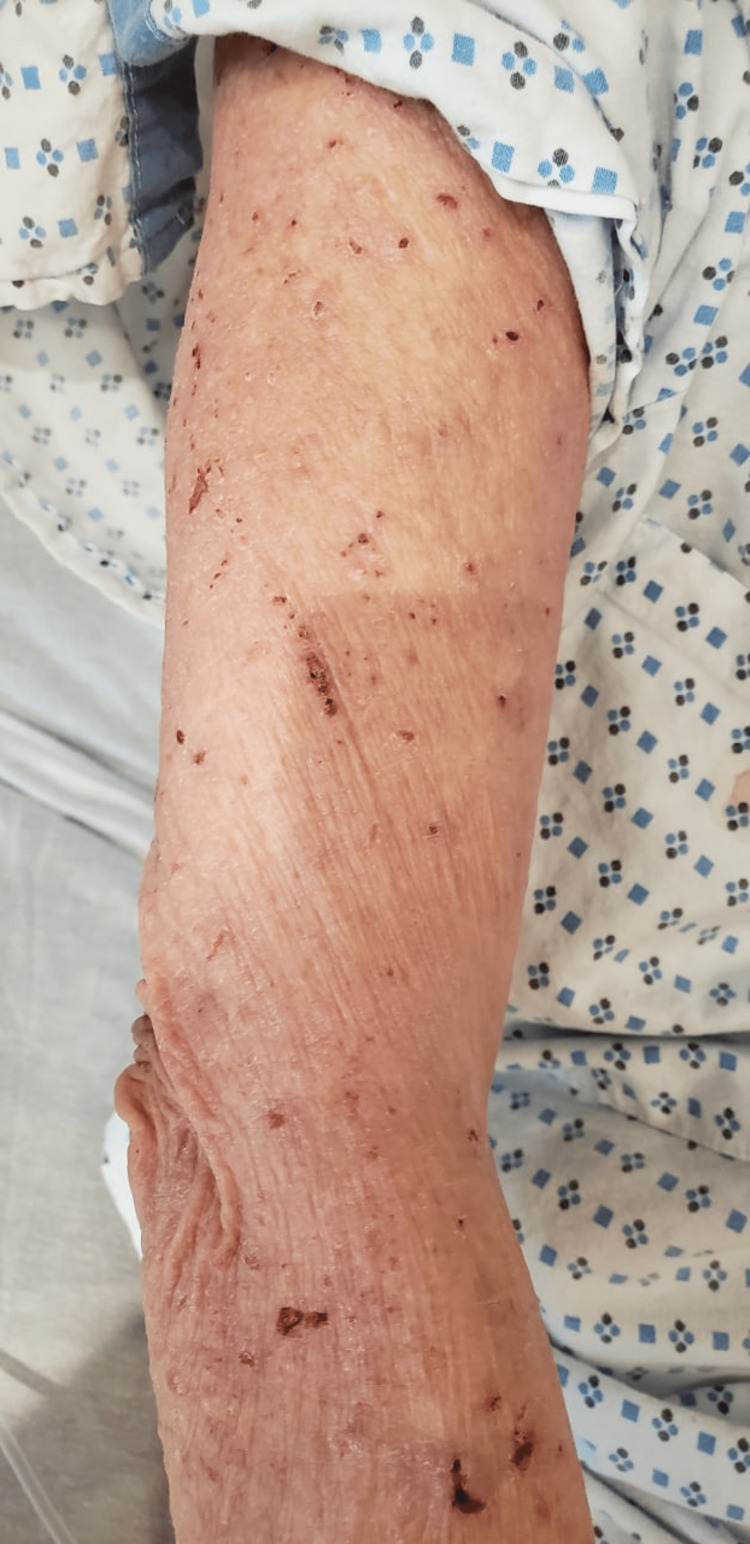
Urticarial papules on the upper extremities

Here, we present a case of an 83-year-old lady with a history of atopic dermatitis, chronic kidney disease (CKD) (baseline creatinine of 2.5 obtained a year prior to presentation), who presented to the hospital on account of generalized erythematous papules and hemorrhagic scabs. The lesions were associated with severe itching and involved the upper and lower extremities and the trunk sparing the hands, feet, and face (Figures 1, 2). The rash started one day after she received her coronavirus 2019 (COVID-19 Moderna) booster dose, however, presented to the hospital six months later due to worsening symptoms. She denied exposure to aggravating factors or using new skin care products. Initially, the patient tried oral prednisone to relieve her symptoms as she used to have specific reactions associated with new medication use; however, the pruritic rash was extensively spreading, worse enough to disturb her sleep and daily functional status, causing altered mentation and necessitating her to present to the emergency department. The patient has no history of malignancy or family history of autoimmune disease but reported rheumatoid arthritis in her uncle and daughter.

Upon presentation, the patient was found to be afebrile (98.5 F), had systolic hypertension (159/72), tachycardic with HR 116 bpm, and saturating at 98% on room air at 20 breaths/min. Laboratory results (Table 1) were significant for leukocytosis of 16.5 x 10(9)/L with an eosinophilic predominance of 21.1%, absolute eosinophils of 3.48 x 10(9)/L, normocytic anemia with hemoglobin of 10.6 g/dL, hematocrit of 32.8%, elevated inflammatory markers including sedimentation rate of 102 mm/hr, C-reactive protein of 15.7 mg/L, creatinine of 3.67 mg/dL, blood urea nitrogen of 75 mg/dL, e-GFR of 12, procalcitonin of 0.71. The rapid COVID-19 antigen test was negative. Electrocardiogram (EKG) showed sinus tachycardia and QTc of 391 ms. Chest x-ray ruled out active pulmonary disease.

**Table 1:** Initial lab evaluation upon admission

Labs	Initial lab findings	Reference Range
Leukocytes (WBC)	16.5 x 10(9)/L (elevated), eosinophils 21.1% (elevated)	3.5-10 (9)/L; Neutrophil:40%-75%; Lymphocyte: 20%-45%; eosinophils 1%-4%
Hemoglobin (Hgb)	10.6 g/dL	13.5-17 g/dL
Hematocrit (HCT)	32.8%	36%-48%
Erythrocyte Sedimentation Rate (ESR)	102 mm/hr	0-15 mm/hr men; 0-20 mm/hr women
C-Reactive Protein (CRP)	15.7 mg/L	<0.3 mg/L
Creatinine (Cr)	3.67 mg/dL	0.7-1.3 mg/dL
Blood Urea Nitrogen (BUN)	75 mg/dL	7-20 mg/dL
e-GFR	12	>60 mL/min
Procalcitonin (PCT)	0.71	<0.1 ng/mL

She received one dose of oral dexamethasone 10mg, diphenhydramine 25mg, and intravenous famotidine 20mg. She was admitted to the hospital where HIV, hepatitis C, urine porphyrin, and anti-nuclear antibodies (ANA) levels were obtained to rule out immunocompromise, porphyria cutanea tarda, and autoimmune disease consecutively. All tests came back negative. The patient was started on an IV antibiotic for sepsis secondary to a possible cutaneous superimposed infection, gentle hydration given her worsening CKD (baseline creatinine was 2.5 years prior to presentation), oral prednisone, and hydrocortisone cream with significant improvement of her symptoms. She was discharged the following day on oral antibiotics and prednisone with a recommendation to follow up with dermatology. Soon after her discharge, the patient had a punch biopsy from the posterior right shoulder rash and was sent for H&E stain and direct immunofluorescence (DIF) assay to further investigate possible autoimmune diseases including but not limited to lupus, connective tissue disease (CTD), and bullous disease. Complete blood work was obtained, including Lyme serology, complement system levels, ANA, rheumatoid factor, lupus antibodies, and inflammatory markers. Meanwhile, the patient was prescribed permethrin 5% for empiric scabies treatment and oral prednisone, hydrocortisone cream, and an oral antihistamine. Two weeks after, the dermatopathology report showed skin with patchy hyper ortho parakeratosis containing serum, acanthosis, spongiosis, and superficial and mild dermal perivascular lymphocytic infiltration with occasional eosinophils. Periodic acid-Schiff stain (PAS) and DIF were both negative. The report signified a spongiotic dermal hypersensitivity reaction with differential including arthropod assault reaction, drug, contact, or allergen related. There was no evidence of scabies or CTD. Labs show ANA 1:80 speckled pattern, ESR <31, SPEP showing high M-spike and alpha-2 globulin, C3 202, C4 15, negative Lyme serology, and dsDNA. The patient was started on Dupilumab subcutaneous injection and oral prednisone 10 mg with significant improvement of her symptoms.

## Discussion

When receiving live attenuated vaccines such as the smallpox vaccine, people with eczema may be at risk of consequences, including eczema vaccinatum. It is unlikely that COVID-19 vaccinations will result in severe skin reactions since it does not include any SARS-CoV-2 live virus. However, there have been numerous reports of local immunologic responses to the COVID-19 vaccines from Pfizer and Moderna, namely, “COVID arm” injection site reactions are believed to be a delayed dermal hypersensitivity response which can peak within three to four days after vaccination. Our report highlights a significantly delayed hypersensitivity reaction in a patient with a history of atopic dermatitis that happened days after the inoculation and peaked six months after. This late timing was consistent with COVID‐19 vaccine‐related delayed hypersensitivity reaction. One of the international studies showed 414 cutaneous reactions to mRNA COVID-19 vaccines from Moderna (83%) and Pfizer (17%). Delayed local reactions were most common, followed by local injection site reactions, urticarial eruptions, and morbilliform eruptions.

Also, it was noted that the Moderna COVID-19 immunization case series exhibited delayed significant local reactions that manifested eight days after the initial dose [4]. However, subsequent investigations report a median onset of seven days after the initial dose [3-5]. These results imply that the late start of skin lesions may be typical in this clinical setting. Furthermore, these symptoms resembled the urticarial eruptions that people with COVID-19 infection experience [6], suggesting that an immunological response to the viral mRNA protein product or cytokines produced by activated immune cells after vaccination may be established. Dupilumab is a monoclonal antibody that acts by inhibiting the IL-4 receptor which is one of the inflammatory markers. it is indicated in moderate to severe atopic dermatitis, and it acts by suppressing the TH-2 immune reaction that could cause the cutaneous symptoms associated with the reaction.

## Conclusions

The new mRNA COVID-19 vaccines may be associated with delayed generalized hypersensitivity reactions. However, reports remain limited, increasing the need to better characterize the incidence, clinical course, and effective treatment. People’s history of eczema should not preclude them from receiving a COVID-19 vaccine that is both safe and effective. Our data support that the skin reactions are minor and self-limited but sometimes can be extensive and require medical management; however, it should not prevent or discourage people from getting the vaccine. Cutaneous reaction symptoms often respond well to treatment, so this should not preclude people from getting the vaccination.

In conclusion, researchers found that COVID-19 vaccines are safe. It was concluded by large studies done before that there is a very small percentage of people who might develop flare of their eczema symptoms following vaccination; however, these reactions are well tolerated with medications including monoclonal antibodies and it should not discourage them from getting the vaccines or boosters.
